# Multi-Center Validation of Artificial Intelligence-Based Video Analysis Platform for Automatic Evaluation of Swallowing Disorders

**DOI:** 10.3390/diagnostics16010045

**Published:** 2025-12-23

**Authors:** Chang-Won Jeong, Dong-Wook Lim, Si-Hyeong Noh, Hee-Kyung Moon, Chul Park, Nayeon Ko, Min-Su Kim

**Affiliations:** 1 STSC Center, Wonkwang University, Iksan 54538, Republic of Korea; mediblue@wkuh.org (C.-W.J.); dwl316@wku.ac.kr (D.-W.L.); nosij123@wku.ac.kr (S.-H.N.); 2 Institute for Educational Innovation, Wonkwang University, Iksan 54538, Republic of Korea; ybnjcw@wku.ac.kr; 3 Division of Pulmonology and Critical Care Medicine, Department of Internal Medicine, Ulsan University Hospital, Ulsan 44033, Republic of Korea; cholssak21@gmail.com; 4 Department of Rehabilitation Medicine, Soonchunhyang University Seoul Hospital, Seoul 05700, Republic of Korea; md.nyko@schmc.ac.kr; 5 Department of Physical Medicine and Rehabilitation, Chungbuk National University Hospital, Cheongju 28644, Republic of Korea

**Keywords:** artificial intelligence, dysphagia, machine learning, rehabilitation, stroke, swallowing, video

## Abstract

**Background:** Videofluoroscopic swallow study (VFSS) is a key examination for assessing swallowing function. Although several artificial intelligence (AI) models for VFSS interpretation have shown high predictive accuracy through internal validations, AI models that have undergone external validation are rare. This study aims to develop an AI model that automatically diagnoses aspiration and penetration from VFSS videos and to evaluate the model’s performance through multicenter external validation. **Methods**: Among the 2343 VFSS videos collected, 309 cases of Q1-grade videos, which were free of artifacts and clearly showed the airway and vocal cords, were included in the internal validation dataset. The training, internal validation, and test datasets were divided in a 7:1:2 ratio, with 2012 images (aspiration = 532, penetration = 932, no airway invasion = 548) used for training. The AI model was developed and trained using You Only Look Once version 9, model c (YOLOv9_c). External validation of the AI model was conducted using 138 Q1 and Q2-grade VFSS videos from two different hospitals. **Results**: According to the internal validation, the YOLOv9_c model showed a training accuracy of 98.1%, a validation accuracy of 97.8%, and a test accuracy of 61.5%. From the confusion matrix analysis, the AI model’s diagnostic accuracy for aspiration in VFSS videos was 0.76 (AUC = 0.70), and for penetration, the diagnostic accuracy was 0.66 (AUC = 0.65). According to the external validation, the AI model demonstrated good performance in diagnosing aspiration (precision: 90.2%, AUC = 0.79) and penetration (precision: 78.3%, AUC = 0.80). The overall diagnostic accuracy of external validation for VFSS videos was 80.4%. **Conclusions**: We developed an AI model that automatically diagnoses aspiration and penetration when an entire VFSS video is input, and external validation showed good accuracy. In the future, to improve the performance of this AI model and facilitate its clinical application, research using training and validation with VFSS video data from more hospitals is needed.

## 1. Introduction

Dysphagia is a subjective symptom of difficulty or abnormalities in swallowing [1]. It is a common symptom in older adults and has been reported to occur in 10–33% of adults over 50 [2], with an associated annual healthcare cost of USD 547 million in the United States [3]. The prevalence of swallowing disorders in the elderly varies depending on the assessment environment, with 51–60% elderly individuals residing in nursing homes experiencing this disorder, 44% of those hospitalized for acute medical conditions, and an average of 15% among elderly individuals living in communities [4,5]. Swallowing disorders can occur in conditions where problems exist with neural control and the structures involved in any part of the swallowing process, such as stroke, Parkinson’s disease, and dementia, which are known as major causes [6]. Nutrition plays a vital role in a patient’s recovery, regardless of the underlying disease, so assessing the precise swallowing function of a patient is an important clinical issue [7].

The videofluoroscopic swallow study (VFSS) is one of the most commonly used tests to assess swallowing disorders and determine dietary options [8]. The patient is given various consistencies of food and liquid mixed with barium, which allows the bolus to be visualized in real-time on an X-ray during swallowing. Because the anatomy and physiology of a patient’s oropharyngeal swallowing function can be assessed clearly through a VFSS, clinicians can offer the patients a safe diet strategy [9]. Based on the VFSS results, patients who cannot swallow dietary bolus or who are at high risk of aspiration pneumonia can be fed through a Levin tube [10,11]. Although the VFSS is a gold standard test for diagnosing dysphagia, clinicians need time to read VFSS images, as these images are derived from a sizeable video clip comprising hundreds of frames [12]. The examiner often reads the recorded clips repeatedly due to the complex anatomy of the neck, and accuracy may vary depending on the examiner’s level of proficiency [13]. Various standardization efforts have been attempted to reduce the poor inter-rater and intra-rater reliability of VFSS, but these methods have not been widely accepted [14].

To overcome this problem, several studies have recently attempted to apply artificial intelligence (AI) to read VFSS images more consistently and precisely [15,16,17,18,19,20]. For example, Lee et al. [20] developed a deep learning model that can detect airway invasion in a completely automated manner, without human intervention. A deep convolutional neural network (CNN)-based classifier was designed to identify the occurrence of airway invasion using a total of 179 swallowing motion videos manually being separated, and this model was reported to achieve 74.2% recall, 97.2% accuracy, and 59.1% precision [20]. Kim et al. [17] applied a CNN to validate a model that classifies VFSS findings as normal swallowing, penetration, and aspiration. Their study showed that the area under the curve for the validation dataset of VFSS images for the CNN model was 0.942 for normal findings, 0.878 for penetration, and 1.000 for aspiration [17]. However, the VFSS is a test that aids in diagnosing swallowing disorders by examining a video consisting of at least 200 frames in real time; alternatively, the examiner can re-observe the video after recording. The previous models required an examiner to select appropriate VFSS test images and diagnosed the presence of a swallowing disorder based on these images, which significantly increased the examiner’s workload in clinical setting.

In our previous research, we developed a system that automatically diagnoses swallowing disorders from the entire VFSS video using AI to overcome this issue [21]. When internal validation was performed, the YOLO model’s diagnostic accuracy for penetration and aspiration was 0.92 and 0.96, respectively [21]. As with previous studies, the results were obtained only through internal validation at a single institution, and there are still limited studies that have validated AI models using multi-center VFSS data through external validation. VFSS protocols vary slightly from hospital to hospital, and the types of food and fluoroscopic imaging methods used also differ across institutions. Even if an AI model performs well at a single institution where it was trained, similar results cannot be guaranteed with data from other hospitals.

Therefore, the purpose of this study is (1) to develop an AI model that can automatically analyze VFSS videos tested at multiple hospitals to assess swallowing function and (2) to externally validate this model and propose an AI diagnostic model for VFSS swallowing disorders that can be utilized in various hospitals.

## 2. Materials and Methods

### 2.1. Study Design and Patients

Between January 2022 and January 2025, a total of 2343 VFSS video clips were collected from the Picture Archiving and Communication System (PACS) at Wonkwang University Hospital. All VFSS examinations were conducted according to the modified version of Logemann’s protocol, which is the standard method for VFSS examinations in hospitals in the Republic of Korea [22]. This retrospective study was performed in line with the principles of the Declaration of Helsinki and was reviewed and approved by the Institutional Review Board (IRB) of the participating hospitals. Due to the retrospective design of the study, the ethics committee waived the requirement for written consent. Clinical and imaging data were anonymized.

### 2.2. Dataset

**Figure 1:**
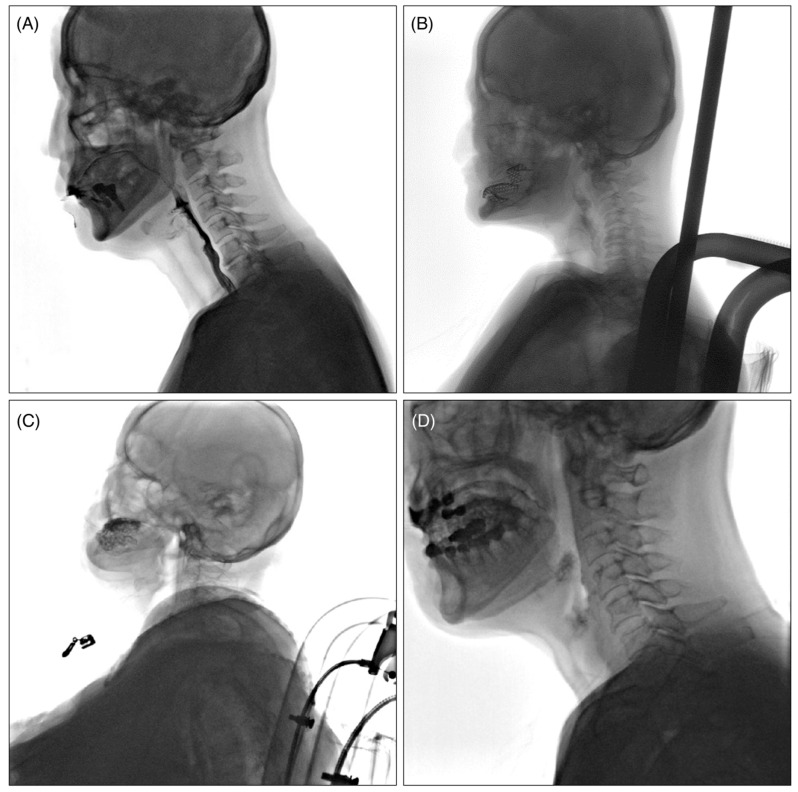
Example of classification according to VFSS video quality. (**A**): Q1; videos in which the airway and epiglottis are clearly visible without artifacts, (**B**): Q2; videos in which the epiglottis is clearly visible but the airway is partially obscured due to the shoulders, (**C**): Q3; videos with severe bolus transit issues during the oral phase, making it difficult to adequately assess the pharyngeal phase, (**D**): Q4; videos with multiple artifacts caused by buttons, previous surgeries, thyroid calcification, constant moving of the patients’ head, etc.

The collected VFSS videos were classified into four levels, Q1 to Q4, according to the quality of the images, with the criteria as follows (Figure 1): Q1: videos in which the airway and epiglottis are clearly visible without artifacts; Q2: videos in which the epiglottis is clearly visible but the airway is partially obscured due to the shoulders; Q3: videos with severe bolus transit issues during the oral phase, making it difficult to adequately assess the pharyngeal phase; and Q4: videos with multiple artifacts caused by buttons, previous surgeries, thyroid calcification, etc.

**Figure 2:**
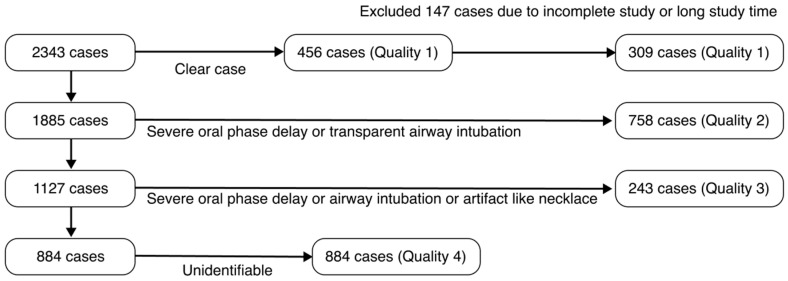
Patient selection flowchart for the training dataset based on VFSS data quality.

Among the 2343 collected video cases, 456 cases were of Q1 high-quality grade. The VFSS analysis pipeline using the object detection AI model required a consistent swallowing process configuration. The following Q1 quality VFSS videos were excluded: (1) cases where the oral, pharyngeal, and esophageal phases in the VFSS video were not fully completed (recorded after the food had already entered the pharynx); (2) cases with excessively long examination times (over 10 min); (3) cases where the starting point of the pharyngeal phase needed for analysis was difficult to identify. A total of 147 cases that were not suitable for AI model training were excluded from this study, and ultimately, 309 cases were included in the development of the AI model (Figure 2).

The internal validation dataset included 309 cases, and the data were divided into training, validation, and test datasets in a ratio of 7:1:2 for analysis. Therefore, 216 cases were used for training, while 31 cases were used for validation and 62 cases were used for the test dataset. A total of 2012 images were used for training to develop the AI model, of which 532 were aspiration images and 932 were penetration images.

To externally validate the dysphagia automatic diagnosis AI model, VFSS video data from 201 cases at Soonchunhyang University Hospital and 10 cases at Ulsan University Hospital were collected. Among the VFSS videos collected from these two hospitals, Q1 and Q2 VFSS videos were used as data for external validation according to the classification in Figure 1. As a result, a total of 138 VFSS video cases (131 cases from Soonchunhyang University Hospital and 7 cases from Ulsan University Hospital) were included in the external validation.

### 2.3. VFSS Diagnostic Process

The VFSS videos were interpreted by three rehabilitation medicine specialists, each with over 15 years of experience in managing swallowing disorders. Each image was classified as ‘no airway invasion’ (no penetration or aspiration), ‘penetration’ (contrast material enters the airway but remains above the true vocal cords), or ‘aspiration’ (contrast material passes below the true vocal cords). Among various test formulas according to viscosity, if aspiration was observed in a single image, it was judged as aspiration, and if only penetration was observed without aspiration, it was judged as penetration. If neither aspiration nor penetration was observed in any of the images, it was interpreted as ‘no airway invasion’. When the diagnostic opinions of the three rehabilitation medicine specialists differed, the VFSS videos were repeatedly reviewed until a consensus was reached. To address potential sources of bias, the specialists responsible for interpretation during the study period were separated from the researchers developing the AI model.

### 2.4. Development of Multi-Frame Medical Image-Labeling Web Application

The proposed system can process high-resolution, multi-frame VFSS videos ranging from 500 MB to 1.5 GB that capture the entire swallowing process from oral intake to esophageal transfer [21]. The uploaded original videos are securely stored via the RESTful STOW-RS (Store Over the Web-RESTful Services) interface, supporting both multi-frame digital imaging and communication in medicine (DICOM) files and standard video formats (e.g., AVI, MP4, MKV).

**Figure 3:**
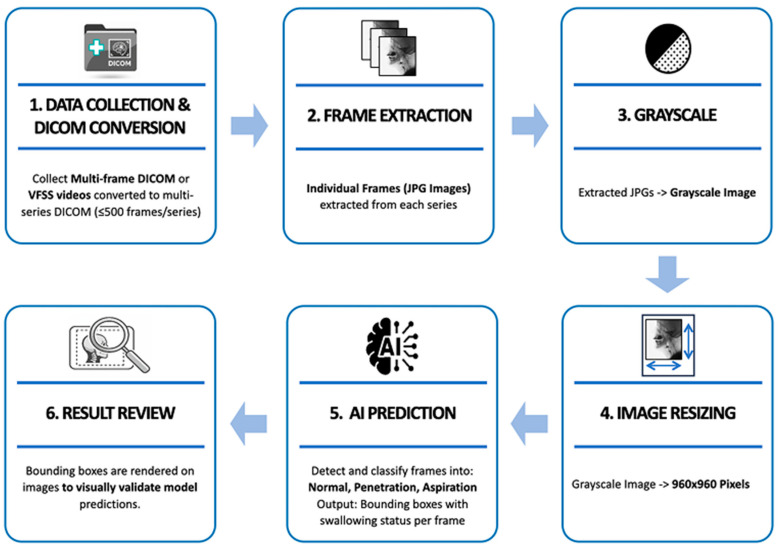
Pre-processing and model prediction flowchart of VFSS multi-frame data.

Each video is broken down into individual frames and converted into a sequential image dataset, which is then used for downstream processing and AI-based prediction. The system is implemented using Python’s Flask micro web framework (version 3.10), providing a lightweight yet scalable architecture. Additionally, it integrates an interactive image-labeling tool that allows clinical experts to annotate swallowing events, as well as a clinical data management module for storing and retrieving patient-specific information. Uploaded videos are stored in a database, and object detection is performed for patient condition assessment by the AI model immediately upon upload. The image analysis process for multi-frame DICOM files or video files captured during VFSS is shown in Figure 3.

**Figure 4:**
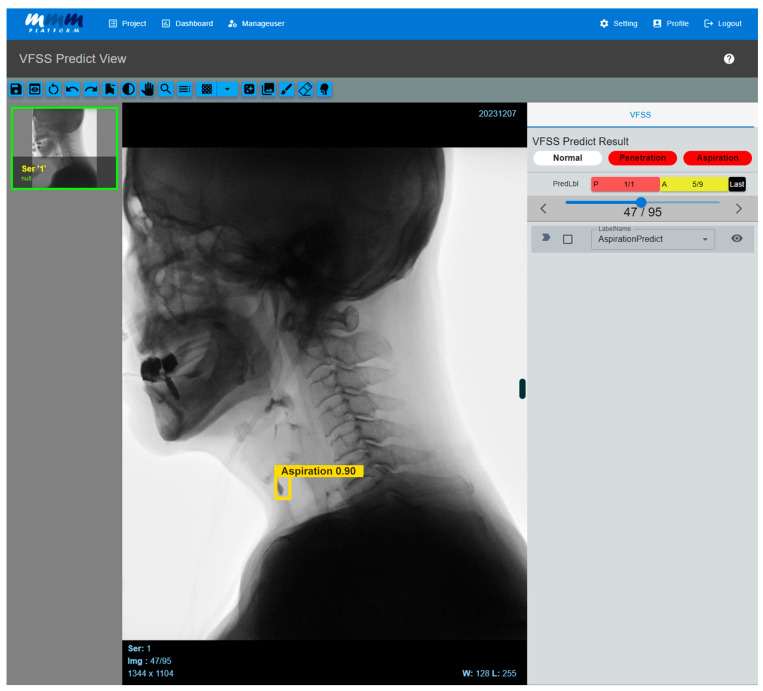
Multi-frame medical image-labeling web application. When a VFSS video is uploaded to an application equipped with the developed AI model, the system automatically analyzes the video and classifies it into penetration or aspiration. If the AI model detects abnormal signs of aspiration, it visually highlights the corresponding frame by displaying a bounding box and confidence value. Users can directly review the AI model’s prediction results, and the system processes the uploaded video frame by frame, providing the detected areas in a time-synchronized manner along with the original video. As a result, clinicians can intuitively identify the exact moments when abnormal findings are detected while playing the video.

When VFSS videos contained a large number of frames due to long recording times, they were divided into multiple series using a self-developed DICOM conversion tool, with a maximum of 500 frames per series. Individual frames were extracted from each divided series as JPG format images, and the extracted images were converted to grayscale to preserve the fluoroscopic characteristics of the videos. Afterwards, they were resized to 960 × 960 pixels to match the model input size. The labeling process was carried out in the same manner as on the previously developed web-based labeling platform [21]. The preprocessed frames were then input into a trained AI model to predict the aspiration, penetration, or no airway invasion. Finally, the prediction results were overlaid on the original video frames in the form of bounding boxes for visual verification and analyzed based on the predicted outcomes (Figure 4).

### 2.5. AI Model Development for Detecting Dysphagia from VFSS

In this study, an AI model was built using the single-stage object detection algorithm You Only Look Once version 9 (YOLOv9). The YOLOv9 architecture generally consists of a backbone, neck, and head. The backbone extracts features from the input image, the neck is designed using a pyramid network approach to combine features extracted from multiple layers of the backbone, and the head performs multi-scale predictions to detect objects at different resolutions based on the combined features [23].

**Figure 5:**
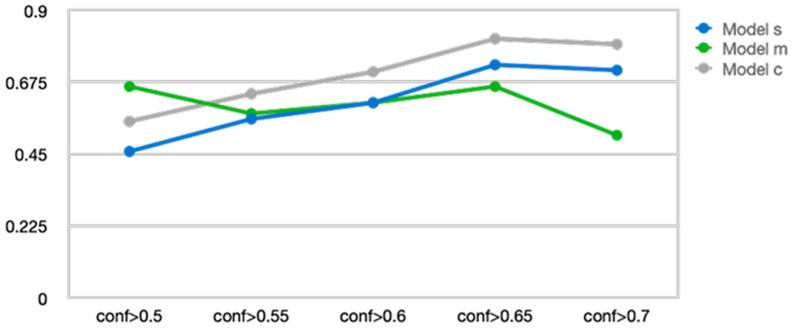
Changes in accuracy according to the confidence level of three You Only Look Once version 9 models.

We compared and optimized the performance of three YOLOv9 variants (Model s, Model m, and Model c) by adjusting the hyperparameters and structural parameters. During the performance validation process, we fine-tuned the model’s confidence threshold and ultimately applied a confidence of 0.65. Confidence refers to the criterion for judging a video as no airway invasion if the prediction score of the object detection model is below the threshold. The performance comparison according to the confidence threshold for each YOLOv9 variant model is presented in Figure 5.

YOLOv9 Model c (YOLOv9_c) demonstrated the most stable and superior performance across all confidence thresholds, maintaining performance without significant degradation even beyond conf > 0.7 after reaching peak performance at conf > 0.65. YOLOv9 Model s (YOLOv9_s) showed gradual performance improvement up to conf > 0.65 but then a slight decline afterward. In contrast, YOLOv9 Model m (YOLOv9_m) exhibited relatively high initial performance in the conf > 0.5 range, but its performance sharply decreased as the confidence increased beyond 0.7. These results suggest that YOLOv9_c is more robust across various confidence criteria, while YOLOv9_m may experience a significant drop in prediction accuracy under high-confidence conditions. Therefore, YOLOv9_c was selected as the most suitable model and was used for all subsequent analyses and applications.

The performance of the AI model was evaluated using precision, recall, mean average precision at 0.5 (mAP@0.5), mean average precision averaged over various intersection over union thresholds (mAP@[0.5:0.95]), receiver operating characteristic (ROC) curves, and area under the curve (AUC) values.

## 3. Results

### 3.1. Internal Validation of the AI Model

Internal validation of the VFSS dysphagia diagnosis model was conducted using a total of 2012 images from 309 cases. The internal validation performance evaluation of this YOLOv9_c model includes precision, recall, mean average precision (mAP), and mean average precision averaged over various intersection over union (IoU) thresholds, as shown in Table 1.

**Figure 6:**
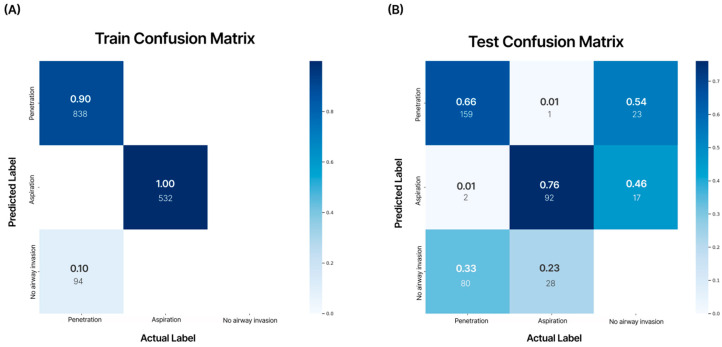
Analysis of the confusion matrix for the training dataset and test dataset. The numbers in each matrix indicate accuracy (large font) and the number of images used (small font). According to the internal validation of the developed AI model, aspiration showed an accuracy of 0.76, and penetration showed an accuracy of 0.66. (**A**) Training confusion matrix; (**B**) test confusion matrix.

The model demonstrated a training accuracy of 98.1%, validation accuracy of 97.8%, and test accuracy of 61.5%. In the confusion matrix analysis, the AI model’s diagnostic accuracy for aspiration on VFSS videos was 0.76 (AUC = 0.70), and for penetration, the diagnostic accuracy was 0.66 (AUC = 0.65) (Figure 6).

### 3.2. External Validation of the AI Model

**Figure 7:**
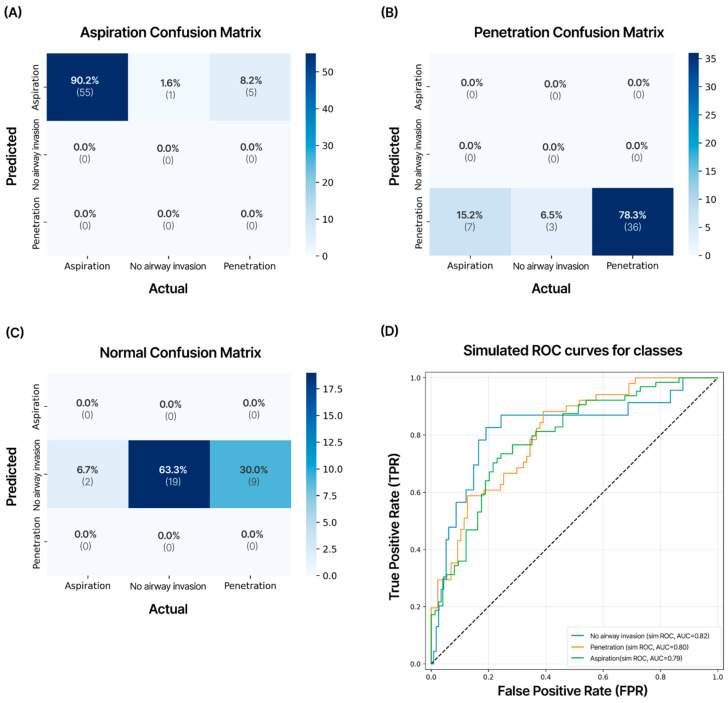
Confusion matrix of the test dataset for external validation. VFSS videos of 138 cases obtained from two other hospitals not included in the development of the AI model were validated. This model showed external validation accuracies of 90.2% for aspiration, 78.3% for penetration, and 63.3% for no airway invasion cases. The numbers in each matrix indicate precision (large font) and the number of cases (small font). (**A**) Aspiration; (**B**) penetration; (**C**) no airway invasion; (**D**) results of receiver operating characteristic (ROC) curves and area under the curve (AUC) values for external validation data on aspiration, penetration, and no airway invasion.

External validation was performed on an AI model developed using 138 cases of Q1 and Q2 quality VFSS examinations conducted at two other hospitals. In the external validation, when experts manually analyzed the VFSS videos, aspiration was diagnosed in 61 patients (44%), penetration in 47 patients (34%), and no airway invasion in 30 patients (22%). The recorded VFSS video file formats consisted of three types—AVI, MKV, and MP4—depending on the hospital and patient and were standardized in the Multi-frame Medical Image-Labeling Web Application in the same manner as DICOM files. The YOLOv9_c model demonstrated strong performance in detecting aspiration (precision: 90.2%, AUC 0.79) and showed good performance in diagnosing penetration (precision: 78.3%, AUC = 0.80) (Figure 7).

The precision for no airway invasion was 63.3%, and the AUC was 0.82, with the overall diagnostic accuracy for all VFSS videos being 80.4%. In the ROC analysis, good AUC values were observed for aspiration.

## 4. Discussion

We developed an AI web application that can automatically diagnose aspiration and penetration, which is crucial for treating swallowing disorders, by analyzing VFSS videos to assess the patient’s swallowing function in a short time. This AI web application can standardize different video formats from each hospital, enabling an automatic diagnosis of swallowing disorders from VFSS examinations conducted at various medical institutions. When performing external validation of the AI model using VFSS videos from two different hospitals, the system demonstrated good accuracy in diagnosing airway aspiration and penetration.

Recently, research has been actively conducted to develop and evaluate AI models as an important tool for automatically interpreting VFSS tests for various phenomena of swallowing disorders, such as airway invasion of food [24], aspiration of food into the airway [15], penetration of food into the airway [21], and measurement of pharyngeal delay time [25]. In particular, checking whether food enters the airway is an important factor in determining whether a patient should receive nutrition through a tube or orally [26]. Initially, studies were conducted to assess the extent of airway invasion, but currently, AI models are being developed that can distinguish airway penetration and aspiration, enabling clinicians to evaluate the severity of a patient’s swallowing disorder accurately [1,26]. Previous studies manually selected a few videos showing swallowing disorders from VFSS videos, which consist of hundreds of frames, for AI training [15,17,24,25,27]. During internal validation, evaluators then chose a few video frames expected to contain abnormalities, rather than the entire video, to assess the accuracy of AI interpretation [15,17,24,25,27].

Kwak et al. [18] developed software that automatically extracts the video frame in which the hyoid bone reaches its highest position and the video frame in which it reaches its lowest position to address the issue of manually selecting frames required for deep learning model development. They conducted a study using the ConvNextTiny CNN model to examine the automatically extracted frames for the diagnosis of aspiration and penetration through VFSS videos, and the model showed good accuracy during internal validation [18]. However, this method has limitations in evaluating patients with various swallowing disorders, including those who have undergone laryngeal cancer surgery, as VFSS frames may be excluded due to differences in airway length and vocal cord position among patients. In this study, to address these issues, we proposed an AI model that can quickly analyze swallowing disorders and is capable of automatically reading swallowing disorders by automatically dividing the entire video into frames to evaluate various patients with swallowing disorders in clinical settings.

The noteworthy aspect of this study is that a model was developed to automatically interpret entire VFSS videos and diagnose airway aspiration and penetration, and external validation was performed using VFSS data from two other hospitals. Previous studies had only conducted internal validation, and research that verifies VFSS AI models using data from external hospitals is rare. Since there are differences in the way VFSS video images are recorded at Wonkwang University Hospital, where AI model training and internal validation was conducted, and at Soonchunhyang University Hospital and Ulsan University Hospital, where external validation was conducted, a program was separately developed to standardize the VFSS video recordings, which was included in the Multi-frame Medical Image-Labeling Web Application. Although VFSS tests vary slightly depending on the country and hospital, they are mostly conducted according to Logemann’s protocol [28], making it easier to standardize video recordings for consistent analysis by AI models [29,30]. In this study, VFSS videos from external validation institutions were excluded from AI training, and only VFSS videos from Wonkwang University Hospital were used. Nevertheless, the YOLOv9_c VFSS AI model demonstrated good diagnostic performance for aspiration and penetration in external validation, suggesting the potential to generalize VFSS AI diagnostic technology.

The characteristics of VFSS videos vary slightly depending on the equipment used at the hospital where they are collected. The VFSS videos from Wonkwang University Hospital, used for internal validation and the Yolov9_c AI model, are saved directly in DICOM format and have a frame rate of 8 frames per second. The videos from Soonchunhyang University Hospital and Ulsan University Hospital, used for external validation, were captured using capture boards. Both hospitals saved their videos at a frame rate of 15 frames per second in mp4 and avi formats, respectively. However, all VFSS video files were converted to DICOM format and processed using the same pipeline. As the pipeline of this study processes one frame (one image) at a time, differences in frame rate did not affect the analysis.

A difference in the performance of the AI model between training and testing was observed during internal validation. The overall precision value was 0.98 for training and 0.61 for testing, showing a difference of 0.37. From the confusion matrix analysis, diagnostic accuracy for aspiration scored 0.76 while that of penetration was 0.66. However, the confusion matrix from external validation testing showed an accuracy of 0.90 for aspiration and 0.78 for penetration. The difference is presumed to be due to a class mismatch in the training data, where the number of training cases for aspiration (532 images) was roughly half of that for penetration (932 images) [31]. Additionally, not using a large-scale VFSS video dataset for training and internal validation may have caused the small size of the test dataset to negatively affect test accuracy during internal validation [32]. Therefore, to significantly improve the VFSS AI model’s performance in diagnosing swallowing disorders, it is necessary to train the model with more videos. In future studies, we plan to collect and train the model with more VFSS video data and apply additional augmentation (flip, rotation, shift, zoom in and out, brightness, etc.) to address the drop in performance and further enhance the model’s performance [33].

This study has several limitations. The AI VFSS video training dataset was collected from a single institution rather than on a large scale. To be applicable to patients across various clinical settings, it is necessary to collect more VFSS videos from multiple medical institutions to sufficiently increase the amount of training data. Although external validation was conducted at two different hospitals, it is necessary to validate VFSS videos from more hospitals. Even though VFSS protocols may be similar across hospitals, the equipment used for filming and video processing settings varies, which can affect the accuracy of VFSS AI models. Additionally, the levels of aspiration and penetration were not quantified. There are evaluation tools that measure the severity of swallowing disorders based on the amount and depth of food entering the airway, and clinicians determine patients’ rehabilitation therapy and safe dietary plans based on the results of these evaluation tools. While an AI model’s diagnosis of aspiration and penetration can indirectly help in deciding a tube diet, for clinical application, it is necessary to present more detailed gradations of aspiration and penetration. These limitations will be addressed in future follow-up studies based on this research.

## 5. Conclusions

In summary, we developed an AI system based on YOLOv9_c that can automatically diagnose airway aspiration and penetration, which are critical for swallowing disorder assessment, from VFSS videos. The advantage of this system is that it can accurately analyze swallowing disorders within seconds by inputting the entire VFSS video, without the need to manually select VFSS images. External validation using VFSS videos from other hospitals showed good accuracy in diagnosing aspiration and penetration. These external validation results suggest that the VFSS AI swallowing disorder diagnostic technology developed by the authors has the potential to be used in evaluating patients with swallowing disorders of various causes across multiple hospitals, regardless of the VFSS video format.

## Figures and Tables

**Table 1:** Internal validation results of an AI model that automatically diagnoses aspiration and penetration in VFSS.

**Cases**	*n* = 309 (Q1 quality) 70% for training (*n* = 216), 10% for validation (*n* = 31), 20% for test (*n* = 62) Wonkwang University Hospital				
**Images for training**	*n* = 2012 Aspiration (*n* = 532), Penetration (*n* = 932), No airway invasion (*n* = 548)				
**Image model**	YOLOv9_c model				
	Confidence: 0.715 for training, 0.876 for test				
	Image input: 960 × 960 pixel				
**YOLOv9_c model internal validation**					
	Precision	Recall	mAP@0.5	mAP@[0.5:0.95]	Images (*n*)
**Training**	0.981	0.900	0.988	0.650	67,426
**Validation**	0.978	0.900	0.986	0.640	6425
**Test**	0.615	0.674	0.615	0.339	22,473

YOLOv9_c, You Only Look Once version 9 model c; mAP@0.5, mean Average Precision at 0.5, mAP@[0.5:0.95]; Mean Average Precision averaged over various Intersection over Union thresholds.

## Data Availability

The datasets analyzed during the current study are available from the corresponding author on reasonable request. The data are not publicly available due to privacy or ethical restrictions.
